# Electronic Screening for Adolescent Risk Behaviors in the Emergency Department: A Randomized Controlled Trial

**DOI:** 10.5811/westjem.2022.7.55755

**Published:** 2022-11-04

**Authors:** Siobhan Thomas-Smith, Eileen J Klein, Bonnie Strelitz, Jennifer Jensen, Elizabeth Parker, Laura Richardson, Carolyn A McCarty, Taraneh Shafii

**Affiliations:** * University of Washington, Seattle Children’s Hospital, Department of Pediatrics, Division of Emergency Medicine, Seattle, Washington; † Center for Clinical and Translational Research, Seattle Children’s Research Institute, Seattle, Washington; ‡ University of Washington School of Medicine, Department of Pediatrics, Division of Adolescent Medicine, Seattle, Washington

## Abstract

**Introduction:**

In this study we aimed to assess the impact of an electronic health assessment with individualized feedback for risk behaviors in adolescents seeking care in a pediatric emergency department (ED).

**Methods:**

We conducted a randomized control trial using a tablet-based screening program with a study population of adolescents in a busy pediatric ED. The intervention group received the screening program with individualized feedback. The control group received the screening program without feedback. All participants received one-day and three-month follow-up surveys to assess behaviors and attitudes toward health behaviors.

**Results:**

A total of 296 subjects were enrolled and randomized. There was no difference in changes in risky behaviors between the control and experimental groups. A higher proportion of participants in the intervention groups reported that the screener changed the way they thought about their health at one-day follow-up (27.0%, 36/133) compared to the control group (15.5%, 20/129, P = .02).

**Conclusion:**

This study successfully tested a multivariable electronic health screener in a real-world setting of a busy pediatric ED. The tool did not significantly change risky health behaviors in the adolescent population screened. However, our finding that the intervention changed adolescents’ perceptions of their health opens a door to the continued development of electronic interventions to screen for and target risk behaviors in adolescents in the ED setting.

## INTRODUCTION

Nearly six million 15–18-year-olds are evaluated in an emergency department (ED) in the United States annually,^1^ and over 8% of 15–17-year-olds rely on the E D for outpatient healthcare visits.^2^ Adolescents who rely on the ED for healthcare have been found to have higher rates of risk behaviors and mental health needs compared to their peers^2^–^7^ and may miss health prevention screening typically completed during primary care visits.

Emergency clinicians recognize the importance and opportunity for screening health risk behaviors in adolescents such as alcohol and drug use and sexual activity. However, existing barriers in the ED setting limit the ability to screen and implement interventions.^7^,^11^ The primary barriers identified include limited time to build the rapport needed to ask sensitive health questions; lack of training in the use of screening tools; concerns that screening may detract from addressing the patient’s chief complaint; perception that screening is not the responsibility of the emergency clinician; and inadequate resources to address the problems that are identified.^7^–^10^

The development of electronic survey technology offers opportunities to efficiently screen adolescents for high-risk behaviors rather than relying on clinician time. Adolescents indicate that they prefer electronic screening over in-person interviews 9,13–16. Electronic screening also offers the opportunity to build in targeted interventions in an individualized manner using internal algorithms. Existing randomized controlled trials of risk-behavior, computer-based interventions with personalized feedback for adolescents in the ED have thus far targeted a singular specific risk behavior as opposed to screening for a range of behaviors. Several of these singular intervention studies have shown promise in reducing risk such as adolescent dating violence^17^,^18^ and alcohol abuse.^18^–^20^ While reducing any risk behavior is desirable, risk behaviors commonly co-occur;^22^–^24^ so, screening for only one risk behavior may be insufficient.

“Check Yourself” is an electronic screening intervention designed to identify and reduce multiple potentially co-existing risky behaviors as outlined by the Bright Futures guidelines,^25^ including alcohol and drug use, depression, sexual activity, and unsafe driving practices. “Check Yourself” also provides electronic feedback to adolescents about their health behaviors, peer behavioral norms, and tips to reduce risk.^26^ In three studies in primary care, the intervention was shown to be associated with increased delivery of risk-behavior counseling, and two of the three studies showed short-term (three-month) reductions in reported risk behaviors among adolescents, while one did not show significant reductions in risk compared to controls although both groups demonstrated risk reductions 27,28.

This randomized controlled trial evaluates the effectiveness of “Check Yourself” in reducing risk behaviors in a population of teens presenting for care in the pediatric ED. We hypothesized the intervention would decrease risky behaviors in adolescents at three-month follow-up compared to usual care.

## MATERIALS AND METHODS

### Study Design

This study was a randomized controlled trial conducted at a pediatric ED between March 2017–December 2018. The ED is part of an urban, tertiary, free-standing children’s hospital that serves a multi-state region with an estimated 50,000 pediatric patients overall and 8,000 adolescent patient visits per year. The study was approved by a hospital institutional review board and was registered in clinicalstrials.gov (Identifier: NCT03304574).

### Population

Adolescent patients (aged 13–18 years) who presented to the ED for care, spoke and read English, and who had an email address or cell phone were eligible for study participation. Exclusion criteria were inability to complete screening due to intellectual disability; acute cognitive impairment due to injury or intoxication; administration of intravenous sedation or pain medications; mental health concern as primary reason for ED visit; or ED visit resulting in hospital admission. Individuals who were admitted to the hospital were excluded from the study due to the potential confounding factors of severity of illness, length of hospital stay, and inpatient, behavioral risk-factor screening on the potential for impacting behavioral change.

**Population Health Research Capsule**

What do we already know about this issue?

*Computer-based interventions for adolescents in the ED can reduce risk for individual behaviors. However, risky behaviors commonly co-occur*.

What was the research question?

*Can a multi-behavior focused electronic health assessment with personal feedback decrease risk in adolescents seeking care in the ED?*

What was the major finding of the study?

*The assessment tool with personal feedback did not decrease risky behaviors, but the tool did change perspectives on health*.

How does this improve population health?

*Visits to the ED are an opportunity for adolescent risk-behavior screening. More work is needed to develop tools that encourage behavior change*.

### The Intervention

All intervention youth completed an electronic health assessment tool with integrated personalized feedback. The tool, “Check Yourself,” was originally designed for use prior to adolescent well-care visits in primary care settings. It takes about 15 minutes to complete and includes recommended screening for key health behaviors based on the Bright Futures guidelines^25^ (eg, alcohol and drug use, depression, sexual activity, driving safety, helmet use, physical activity, and nutrition). The tool provides integrated, individualized feedback based on motivational strategies such as normative feedback (comparison to peer behaviors and health guidelines); information regarding potential consequences of behaviors; and practical tips to change behavior.

At the completion of the feedback component of the tool, adolescents were given the option to receive additional online informational resources by email. In two of the three studies conducted in a primary care setting, the tool has been well received by adolescents and healthcare clinicians; and has shown to be associated with short-term (three-month) reductions in reported risk behaviors among adolescents.^26^,^27^ In the primary care studies, clinicians also received a one-page printed summary of adolescent-reported risk behaviors alerting the clinician to areas of high and moderate risk to encourage in-clinic counseling. Due to the fast-paced workflow in the ED, results of the risk screening were not provided to the emergency clinician. Instead, the adolescent patient only received integrated feedback with the tool.

### Control Group

The control group received electronic screening only using a similar electronic screening interface. Control youth did not receive any individualized feedback.

### Procedures

Prior to study enrollment, a simple randomization sequence was prepared by a statistician with no clinical involvement in the study with 300 potential allocations per arm. Once enrollment opened, clinical research coordinators (RC) prospectively identified adolescents during their ED visit and invited the patients to participate in the study. After verifying eligibility, adolescents <18 years provided assent and their caregivers gave consent. Adolescents who were 18 years old consented for themselves. After obtaining consent, RCs then used the REDCap (Research Eectronic Data Capture) computer randomization module to allocate participants to the control or intervention arm of the study. The RCs were present in the ED to enroll subjects seven days a week from 1 pm – 11 pm.

To ensure privacy while adolescents were using the computer tablets, caregivers were instructed not to view the tablet or participate in the screening questions. The RCs instructed adolescents not to discuss or share their responses with caregivers. As a safety measure, a flagging system was enacted to promptly notify the ED clinician (at baseline) or study clinicians (at follow-up) when an adolescent endorsed suicidality on the depression screen, regardless of study arm assignment. At baseline, ED procedures for suicidal patients included an evaluation by the ED attending who determined need for further evaluation. If further evaluation was deemed necessary, a mental health professional assessed the participant while in the ED and prior to discharge. For follow-up surveys, study clinicians called participants and conducted a phone interview to assess safety and ensure appropriate follow-up care. This protocol was implemented in the same manner across study arms.

All participants (intervention and control) were asked to complete online follow-up surveys at one day and three months after their initial ED visit. Follow-up periods of one day and three months were chosen due to similar follow-up periods with previous trials of the Check Yourself tool. Online follow-up surveys were collected using REDCap. (REDCap at the University of Washington Institute of Translational Health Sciences (ITHS) is supported by the National Center for Advancing Translational Sciences of the National Institutes of Health under Award Number UL1 TR002319.) Participants were invited and reminded to complete the survey via short text message notifications, sent by an automated text messaging service (Twilio Inc, San Francisco, CA). The one-day follow-up survey asked adolescents whether the screening and feedback tool had changed the way they thought about their health. The three-month follow-up survey included a reassessment of the same risk behaviors assessed at baseline. Participants received a $10 gift card for each completed survey (up to $30 total).

### Statistical Analysis

We used data on brief intervention effects with adolescents from the existing literature (Ozer 2005; Patrick 2001; Werch 2011) to conduct power calculations with PASS 11 (NCSS Statistical Software, Kaysville, UT), assuming two-sided statistical tests and *P* = 0.05. Based on inequality tests for repeated measures designs across means with a within-subject correlation (rho) = 0.5, a sample size of 150 per arm achieves >0.90 power to detect a difference in mean change of one point in risk-behavior summary.

### Measures

#### Overall Risk Score

We calculated an overall risk score based on 13 risk behaviors screened for by the electronic tool including risks ranging from sleep behaviors and exercise to driving under the influence and inconsistent condom use. Ratings for risk behaviors were determined a priori and are consistent with prior studies of this tool.^27^ We defined high-risk variables as those causing imminent harm such as driving under the influence and were assigned a risk score of 2. Moderate risk variables defined as those that impair health over time but not associated with risk for short-term morbidity or mortality, such as lack of exercise, were assigned a risk score of 1.

### Individual Behaviors

#### Depression

We used the Patient Health Questionnaire 2-item to assess for depressive symptoms, using the questions: “Over the last two weeks how often have you been bothered by having little interest or pleasure in doing things?” and “Over the last two weeks how often have you felt down, depressed or hopeless?”^32^

#### Substance Use

Variables for substance use included marijuana and alcohol frequency of use over the prior 30 days. Alcohol frequency was calculated by number of days and number of drinks per day. (One drink = one can/bottle of beer, one shot of liquor, one glass of wine).

#### Sexual Behavior

Sexual behavior risk was a composite variable of frequency of condom and/or birth control use with sex (Table 1).

### Perception of Screener

At one-day post visit follow-up participants were asked if the screener changed the way they thought about their health. We included this variable to further assess the perceived impact of the screening and intervention tool in the ED.

### Data Analysis

R version 3.6.3 (R Foundation for Statistical Computing, Vienna, Austria) was used for data analysis. We calculated means and standard deviations and conducted bivariate analyses to examine demographic differences (age, race, and gender identity) between the control and intervention groups. Chi-square tests were used for categorical variables and a t-test for continuous variables. The individual risk-behavior variables were constructed, and percentages of risk/no risk for each variable were calculated by treatment group and time period (baseline or three months). We used linear regression to assess the effects of the intervention on risk behaviors from baseline to three-month follow-up.

We conducted exploratory analyses to assess the intervention effects on specific risk behaviors included in the composite risk variable, such as substance use and inconsistent condom use. In addition to the main outcome measure, we examined the impact of the intervention on individual behaviors. These are behaviors that were deemed to be more acutely impactful on morbidity and mortality in this age group. Definitions for how risk was defined for each of these individual variables is provided below. We used binomial logistic regression for categorical variables and linear regression continuous variables. The control group was the reference group for all statistical models with age, gender identity, and baseline risk included as covariates.

## RESULTS

**Figure 1:**
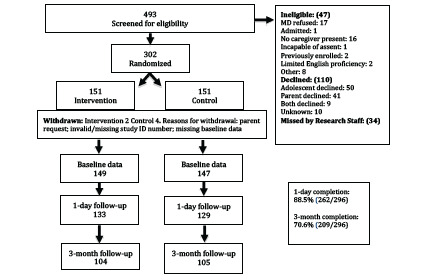
Flow diagram of study participant enrollment, allocation, follow-up and analysis. *MD*, Doctor of medicine; *ID*, identification.

A total of 412 of 493 participants approached were determined eligible for the study. Of those eligible, 296 joined the study, ultimately yielding an acceptance rate of 71.4%. The sample was comprised of 147 adolescents in the control arm and 149 in the intervention arm (Figure 1). The retention rate was 89.1% (262/294) at the one-day follow-up and 71.1% (209/262) at three-month follow-up. The sample consisted of 53% females with a mean age of 15.2 years. Of note, there was a difference in age between the groups (t = 2.44, *P=*.02) with the control group being significantly older (mean = 15.4 years) than the intervention group (mean =15.0 years) (Table 2).

### Overall Risk Score

Prevalence of individual risk behaviors used to create the overall risk variable are presented in Table 1. The overall risk score mean at baseline was 5.87 (SD = 3.66) in the control group and 4.79 (SD = 3.66) in the intervention group. At three-month follow-up the overall mean risk score was 5.96 (SD = 3.43) for the control group and 4.42 (SD = 3.41) for the intervention group. Controlling for age, gender, and baseline risk score, we found no significant difference in reduction of risk for the intervention group compared to the control.

### Individual Risk Behaviors

In an exploratory analysis of individual risk behaviors, there were no differences found between control and intervention groups at three-month follow-up for depression, marijuana use, alcohol use, or sexual behavior risk (Table 1).

### Perception of Screener

A higher proportion of participants in the intervention groups reported that the screener changed the way they thought about their health at one-day follow-up (27.0%, 36/133) compared to the control group (15.5%, 20/129), controlling for age and gender (odds ratio 2.12; 95% confidence interval 1.14 – 4.03; *P =*.02).

## DISCUSSION

This randomized clinical trial tested an electronic health assessment with individualized feedback for risk behaviors in adolescents seeking care in a pediatric ED. This study is unique as it was a large, randomized trial of a brief, multi-risk eHealth intervention with individualized feedback for adolescents in the ED. Although we found no difference in reduction of overall risk score between intervention and control groups at three months, participants reported the intervention changed the way they thought about their health.

The “Check Yourself” tool was first tested in primary care settings where primary care physicians were provided a print-out of their patient’s risk behaviors to facilitate discussion of preventive health at the visit.^27^ The setting for our study was a busy, fast-paced ED where clinicians focused on addressing the chief complaint and not on discussing preventive health. The emergency clinicians were not provided a print-out of risk behaviors nor were they expected to address health prevention topics; thus, this study in effect tested the brief eHealth feedback as a stand-alone intervention.

To assess for the intervention’s impact on risk behaviors that may be more commonly encountered in the ED, we performed an exploratory analysis on the outcomes of risk for marijuana use, alcohol use, depression, and risky sexual behavior. These risk behaviors were not decreased in the intervention group compared to the control group at three-month follow-up. There was a significant difference between groups at one-day follow-up with more intervention participants reporting that the “Check Yourself” tool impacted the way they thought about their health than those in control group, indicating a perceived attitudinal shift that merits further study.

Most risk behavior intervention studies in EDs target a single risk behavior,^7^ whereas the intervention in this study targeted 13 health behaviors. This may have diffused the impact on any one behavior affecting health. In addition, adolescents in the ED may be preoccupied by their reason for seeking care and less invested in learning about risks that are secondary to their presenting concerns. However, the literature supports that adolescents are open to risk-behavior screening in the ED regardless of their chief complaint. Studies have found acceptability for both specific risk behaviors such as substance abuse;^15^ pregnancy prevention;^33^ sexually transmitted infection risk;^34^–^36^ depression;^37^ suicidality;^38^ and for comprehensive screening across a battery of five risk behaviors (substance use, violence, depression, human trafficking, and access to firearms).^39^

Risk behavior screening in the ED is an important tool for adolescent health as it can reach a population that does not frequently access preventive healthcare. Such screening has increased the identification of substance abuse, post-traumatic stress, depression, and suicidality.^40^–^43^

Similar to our intervention, several studies have included brief, targeted interventions for behavioral change specifically for adolescent dating violence^17^–^18^ and alcohol abuse.^18^–^20^ Unlike these studies, however, our intervention assessed and provided feedback on a wide variety of behavioral risk factors, rather than more streamlined singular behaviors or areas as targets. As the screening was broader, the intervention itself required brevity to fit the time constraints of an ED visit. The difference in outcomes of our electronic screening and intervention tool compared to more focused interventions suggests that the use of multi-variable screening and feedback may not be as successful of an intervention on youth behaviors as targeted screening and feedback focusing on one achievable goal.

## LIMITATIONS

This study has several limitations. The intervention targeted health behaviors with both long-term implications and those with more immediate health consequences. Overall, our study population had low prevalence of risky behaviors compared to the general population screened in the national Youth Risk Behavioral Surveillance System (YRBS) with the exception of depression, which was similar to the YRBS.^21^ These prevalence differences may limit the generalizability of the study. Youth in the ED may have been more concerned about the reason for their acute visit rather than those behaviors addressed in the intervention. Unlike the primary care trial of the “Check Yourself” tool, this intervention did not include discussion with a healthcare clinician, and thus may not have had as much impact. While there was a significant finding of the intervention impact in how adolescents perceived their health, there were no follow-up questions to understand the specifics on how their beliefs changed.

## CONCLUSION

This study successfully tested a multi-variable electronic health screener in a real-world setting of a busy pediatric ED. We were able to implement screening and feedback for health behaviors into typical ED workflow without adding to the workload of clinicians. The “Check Yourself” tool did not significantly change health behavior risks in the adolescent population screened. However, based on our one-day follow-up, our intervention did show an impact on how adolescents perceive their health, opening a door to the continued development of electronic interventions to screen for and target risk behaviors in adolescents in the ED setting. Future studies should focus interventions designed for specific risk behaviors with more depth that could result in more immediate healthy changes and health consequences.

## Figures and Tables

**Table 1:** Risk behavior change at baseline and three months for control and intervention groups.

	Control Group n (%)		Intervention Group n (%)		P-value

	Baseline	At 3 mos follow	Baseline	At 3 mos follow	
	N = 147	N = 105	N = 149	N = 104	
Nutrition					
Low fruit/vegetable intake 0–3/day	113 (76.9)	69 (65.7)	119 (80.0)	81 (77.9)	.12
High sugary drinks >2/ day	59 (40.1)	46 (43.8)	40 (26.8)	28 (29.6)	.057
Activity					
Low sleep time <8 hours/night	81 (55.1)	66 (62.8)	61 (40.9)	42 (40.4)	.0351
Low physical activity 0–3 days/week	55 (37.4)	45 (42.8)	41 (27.5)	28 (26.9)	.72
Safety					
Inconsistent seatbelt use	28 (19.0)	16 (15.2)	21 (14.1)	14 (13.5)	.43
Inconsistent bike helmet use	89 (60.5)	52 (49.5)	79 (53.0)	38 (36.5)	.11
Ever drives drunk or high	4 (2.7)	2 (1.9)	2 (1.3)	2 (1.9)	NA2
Ever texts while driving	22 (15.0)	16 (15.2)	19 (12.8)	18 (17.3)	.18
Drugs and Alcohol					
High alcohol use	28 (19.0)	14 (13.3)	19 (12.8)	10 (9.6)	.41
High marijuana / other drug use	33 (22.4)	20 (19.0)	22 (14.8)	13 (12.5)	.87
Any tobacco Use	14 (9.5)	12 (11.4)	10 (6.7)	7 (6.7)	.23
Sexual behavior					
Inconsistent birth control/ condom use	23 (15.6)	14 (13.3)	19 (12.8)	6 (5.8)	.0461
Depression					
High PHQ-2 score >=3	49 (33.3)	35 (33.3)	43 (28.8)	31 (29.8)	.84

1 Statistical significance set at P<.05.

2 NA – statistical tests not performed if baseline data for intervention and control group n<10

PHQ-2, Patient Health Questionnaire 2-item.

**Table 2:** Demographic characteristics of study population.

	Control	Intervention	Total

	N = 145^1^	N = 149	N = 294
Age, mean (SD)	15.4 (1.6)	15.0 (1.5)^2^	15.2 (1.6)
Gender, n (%)			
Female	75 (51.7)	81 (54.4)	156 (53.1)
Male	70 (48.3)	67 (44.9)	137 (46.6)
Other	0	1 (0.7)	1 (0.3)
Race/Ethnicity, n (%)			
White	79 (54.5)	90 (60.4)	169 (57.6)
Multiracial/other	32 (22.1)	27 (18.1)	59 (20.1)
Black	16 (11.0)	10 (6.7)	26 (8.8)
Hispanic	13 (9.0)	7 (4.7)	20 (6.8)
Asian	3 (2.1)	10 (6.7)	13 (4.4)
Hawaiian / Pacific Islander	2 (1.4)	4 (2.7)	6 (2.0)
Native American	0 (0.0)	1 (0.7)	1 (0.3)

1 N = 147 with 2 participants missing demographic data.

2 P<0.02.
